# Increase of *Neisseria meningitidis* W:cc11 invasive disease in Chile has no correlation with carriage in adolescents

**DOI:** 10.1371/journal.pone.0193572

**Published:** 2018-03-08

**Authors:** Paulina S. Rubilar, Gisselle N. Barra, Jean-Marc Gabastou, Pedro Alarcón, Pamela Araya, Juan C. Hormazábal, Jorge Fernandez

**Affiliations:** 1 Sub-Department of Molecular Genetics, Biomedical Department, Public Health Institute, Santiago, Chile; 2 Pan American Health Organization/ World Health Organization, Washington, D.C., United States of America; 3 Bacteriology section, Infectious Diseases Sub-Department, Biomedical Department, Public Health Institute, Santiago, Chile; 4 Infectious diseases sub-Department, Biomedical laboratory department, Public Health Institute, Santiago, Chile; Instituto Butantan, BRAZIL

## Abstract

*Neisseria meningitidis* is a human exclusive pathogen that can lead to invasive meningococcal disease or may be carried in the upper respiratory tract without symptoms. The relationship between carriage and disease remains poorly understood but it is widely accepted that decreasing carriage by immunization should lead to a reduction of invasive cases. Latin America has experienced an increased incidence of serogroup W invasive cases of *Neisseria meningitidis* in the last decade. Specifically in Chile, despite low total incidence of invasive cases, serogroup W has become predominant since 2011 and has been associated with elevated mortality. Expecting to gain insight into the epidemiology of this disease, this study has used molecular typing schemes to compare *Neisseria meningitidis* isolates causing invasive disease with those isolates collected from adolescent carriers during the same period in Chile. A lower carriage of the serogroup W clonal complex ST-11/ET37 than expected was found; whereas, the same clonal complex accounted for 66% of total invasive meningococcal disease cases in the country that year. A high diversity of PorA variable regions and fHbp peptides was also ascertained in the carrier isolates compared to the invasive ones. According to the results shown here, the elevated number of serogroup W invasive cases in our country cannot be explained by a rise of carriage of pathogenic isolates. Overall, this study supports the idea that some strains, as W:cc11 found in Chile, possess an enhanced virulence to invade the host. Notwithstanding hypervirulence, this strain has not caused an epidemic in Chile. Finally, as genetic transfer occurs often, close surveillance of *Neisseria meningitidis* strains causing disease, and particularly hypervirulent W:cc11, should be kept as a priority in our country, in order to prepare the best response to face genetic changes that could lead to enhanced fitness of this pathogen.

## Introduction

*Neisseria meningitidis* (NM) is an aerobic Gram-negative diplococcus. This human pathogen is a member of the *Neisseriaceae* family and can cause localized or invasive infections. Thirteen serogroups have been defined, of which serogroups A, B, C, W, and Y have accounted for almost all invasive meningococcal disease (IMD) outbreaks worldwide [1,2]. Transmission of NM occurs through direct contact with respiratory secretions or saliva droplets from either infected or asymptomatic colonized individuals (carriers), and possibly by sharing of vessels, where this bacterium has been reported to survive up to 10 days [3].

IMD rates are extremely high in countries belonging to the so called “meningitis belt” in sub-Saharan Africa, where seasonal epidemics occur at rates of 10–1,000 cases/100,000 population [1]. Other countries have experienced epidemics such as the case in New Zealand that reached an incidence of 16.7/100,000 in 2001 [4]. Outbreaks in the meningitis belt are usually dominated by specific lineages of NM as temporal waves. Namely, confirmed cases of IMD in this region were mostly caused by NM serogroup A throughout years 2004 to 2009 and then by serogroup W between 2010 and 2013 [5] with localized epidemics of NM serogroup X [6]. Currently the meningitis belt is dominated by IMD cases associated with serogroups W and C [7] with special concerns presented by the increase of this last serogroup [8]. Countries with lower incidences have been rather dominated by a specific serogroup: Serogroup B constituted 74% of IMD cases between the years 2004 and 2014 in several EU/EEA countries [9], the same serogroup dominated at least between 2000–2007 in Sweden [10], and predominated in West Australia between 2000 and 2014 [11]. IMD incidence in Chile is low (<0.6%/100,000 population), which is currently caused mainly by serogroup W and followed by serogroup B of NM [12–15]. Due to the elevated mortality rates of this strain, in 2012 the Ministry of Health implemented an immunization program with a tetravalent vaccine for all children aged 9 months to 5 years and maintained thereafter for 1-year old children.

Carriage of NM, however, has shown a high diversity of NM lineages and rates worldwide. As a general approximation, cross-sectional studies estimate carriage ranging from 10 to 35% [1,16]. Studies that have assessed the prevalence of carriage within specific countries/populations show remarkable differences. For instance, in a study carried out on university students in the UK during the winter of 2009/2010, it was found that carriage rates could reach 55% [17]; another recent study in the UK shows carriage rates from 3.9 to 26.5% in students aged 10–25 [18]; while carriage in children and young adults in The Philippines ranged, according to age, from less than 3% to 9% [19]. In Chile, a previous study performed in university students has revealed a lower carriage rate compared to European countries (4% in a cohort of 500 students] [20], a result that was attributed to our educational system lacking massive student residences. In a more recent study, the overall carriage observed reached 6.5% (CI: 5.7–7.3%) [21]. A meta-analysis made by Christensen et al. has established, after searching and comparing different studies worldwide, that carriage rates peak at 19 yrs (average carriage of 23%), and subsequently decrease into adulthood [22]. Although numerous studies coincide with higher carriage rates of NM in adolescents, IMD is especially frequent in infants [6] due to an undeveloped immune system and a declined passive immunity from the mother [1]. However, only a few studies have assessed carriage in children. For example, Gold et al., estimated carriage rate in children of the US as 0.71% in the first four years of life [23]. In Chile, one study published during the early ‘80s reported 10% carriage in children between 0–4 years and 15% carriage at ages 5–9 years [24]. To date, there is no carriage data available from adults nor elderly people of the country.

Some IMD outbreaks and hyperendemics have coincided with an increased NM carriage within populations [25–27]. Although outbreak strains have often been found as the dominating ones among carriers, it is not possible to make a proportional correlation and exceptions have also been found [28,29].

To evaluate the increased incidence of serogroup W in Chile, the Public Health Institute of Chile carried out a study to determine the prevalence of nasopharyngeal carriage in children and adolescents aged 9 to 19 years [21]. The aim of the present study was to characterize and compare NM isolates collected in Chile during the year 2013 from children/adolescent carriers (184 samples) and from invasive disease cases (119 samples). Sequencing of MLST genes, as well as PorA and fHbp (these two proteins are currently used, together with other immunogens, in some vaccine formulations) was accomplished for every isolate. We hypothesized that we could find a genetic relationship amid these groups of isolates that could explain the emergence of serogroup W of NM in our country. It is expected that this data could also provide useful information for public health authorities in Chile and abroad.

## Materials and methods

### Samples

*Neisseria meningitidis* isolates from 184 healthy carriers and 119 from patients with invasive disease were obtained from the national reference laboratory at the Public Health Institute of Chile, which receives samples from the whole country as part of the *N*. *meningitidis* surveillance program. Carrier isolates were obtained from children 9–19 year olds in the regions that concentrate most of IMD cases (Metropolitana, Valparaíso and Bio-Bío) between April and June of 2013 as previously described [21]. Informed written consent forms were required from subjects 18 and older. Whereas informed assents were given to subjects under 18 in addition to an informed consent form fulfilled by a legal guardian/parent. The present study, as well as consent/assent forms, were approved by the corresponding ethics committee “Comite de Etica de la Investigacion-Servicio de Salud Metropolitano Norte" Approval date: March 11 of 2013.

### Multilocus Sequence Typing (MLST)

DNA extraction was performed directly from cultured bacteria using thermal shock or using QIAamp DNA minikit (Qiagen) according to the manufacturer’s instructions. The lysate and DNAs obtained were used for polymerase chain reaction (PCR) amplification using the Veriti ™ 96-well Thermal Cycler Applied Biosystem, the MLST primers described for on the website (https://pubmlst.org/neisseria/info/primers.shtml) and GoTaq® Green Master Mix 2X (Promega), with a few modifications in cycling compared to the suggested protocol to get the best results with our enzymes and equipment (S1 Table). Sequencing was performed as previously described [14] using an Applied Biosystem™ 3500 Genetic analyzer. For designation and incorporation of new Sequence Typing (ST) or Clonal Complex (CC) we used the PubMLST databases (http://pubmlst.org/neisseria/).

### PorA and fHbp

Cultured bacteria lysates or DNAs were used for PCR amplifications of PorA and fHbp genes as previously described [13,14] with a few modifications in cycling detailed in S1 Table. Purified PCR products were sequenced using primers previously published [30] and the Applied Biosystem™ 3500 Genetic analyzer. Respective variable regions and alleles were assigned from the same database **(**http://pubmlst.org/neisseria/).

### Statistics

Graphs and statistics were performed using GraphPad Prism5 software; whereas minimum spanning trees were performed using Bionumerics software version 6.6.

## Results

### Distribution by age of NM isolates 2013

**Fig 1:**
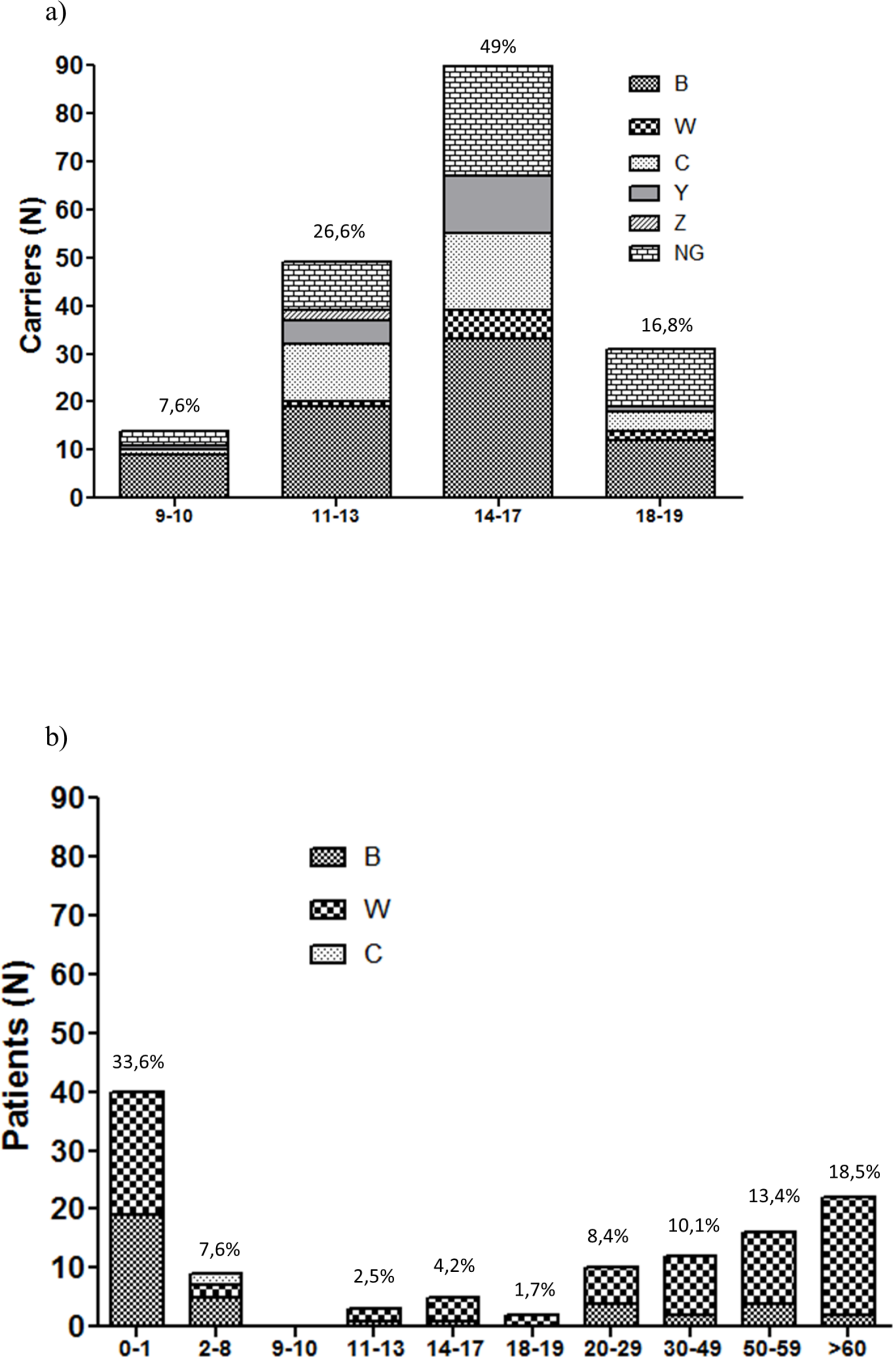
Distribution of NM isolates according to age. All isolates were grouped by age and serogroups were stacked within bars with different patterns from a) carriers (N = 184, 9–19 year olds) or b) IMD (N = 119, any age).

The distribution of carrier isolates by age (Fig 1), showed a diverse representation of serogroups, with serogroup B and non-groupable isolates as the more frequent isolates, followed by serogroups C and Y (Fig 1A).

When distributed by age, invasive isolates were especially concentrated within the first year of life (Fig 1B), followed by a reduction of cases and an increased incidence over 50 years. Serogroup W was the dominant serogroup at all ages, excepting in the group of 2–8 year olds where it was reduced. On the other hand, serogroup B was found at all ages among invasive isolates at different percentages (Fig 1B).

### Distribution of NM isolates by serogroup and clonal complex

**Fig 2:**
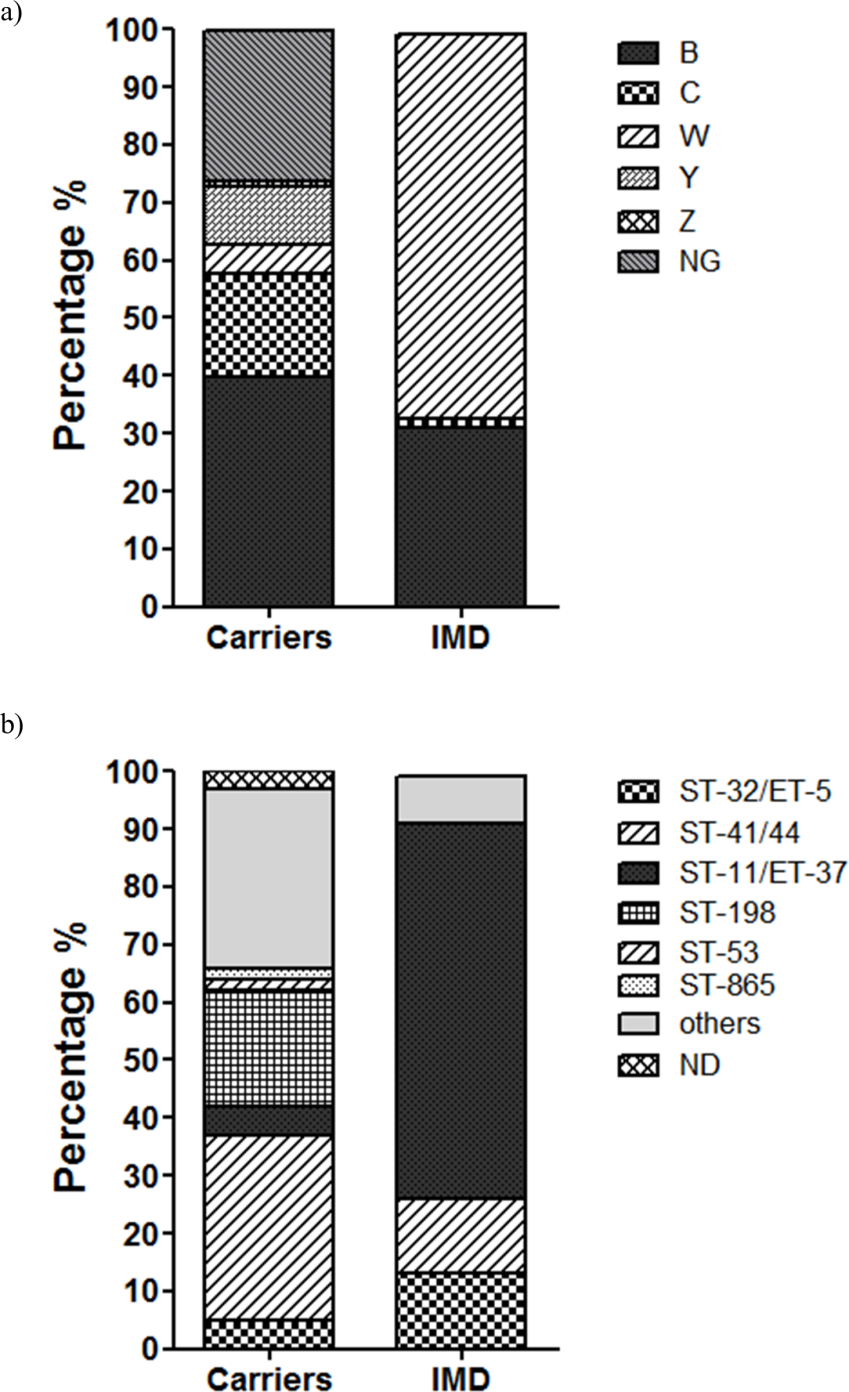
Distribution of NM isolates 2013 by serogroups and clonal complexes. a) Isolates arranged by serogroup percentages show diversity among carriers whereas serogroups W and B account for almost all IMD isolates **(**N.G. = non-groupable). b) Isolates arranged by clonal complexes percentages resulting from MLST analysis presented a separation of serogroup B, both carriers and IMD isolates, into cc41/44 and cc32. Most NG isolates belonged to cc198, whereas all serogroup W isolates belonged to cc11. (Total number of samples: Carriers = 184 (9–19 yrs); IMD = 119 (any age)).

When samples were analyzed independent of age, IMD and carrier isolates presented several differences. For instance, comparison of serogroup distribution (Fig 2A) shows that serogroup W was strongly associated with invasive disease (66% within invasive isolates and 5% within carriers). Conversely, non-groupable isolates were rather associated with carriage (26% among carriers and absent among invasive isolates). Likewise, serogroup C seemed to be associated with carriage, reaching 18% among carrier isolates, and less than 2% among invasive isolates. Finally, similar percentages of isolates belonging to the serogroup B (32% and 40% within invasive and carrier isolates respectively) were found.

After MLST analysis, isolates were grouped by clonal complex as shown in Fig 2B. Among carrier isolates we found a higher diversity of clonal complexes (Fig 2B, left bar). Namely, the clonal complex ST-41/44 (hereafter cc41/44) was found to be predominant followed by “others” with similar percentages. Additionally, 20% of carrier isolates belonged to the clonal complex ST-198 (hereafter cc198); whereas this clonal complex was almost absent among invasive isolates. The clonal complexes frequently found among invasive isolates such as ST-11/ET37 and ST-32/ET-5 (hereafter cc11 and cc32 respectively) were similarly present at 5% each among carrier isolates. Interestingly, the group “others” was mainly constituted by isolates without clonal complex assignments (around 25% of total isolates from carriers) which represent isolates that have not previously been reported. On the other hand, a few isolates from carriers were noted as ND (not determined) as we could not amplify one or more MLST genes and thus they could not be associated to any sequence type on the international database.

The IMD isolates were dominated by the hypervirulent cc11 (all belonging to serogroup W) reaching 66%, followed by cc32 and cc41/44 clonal complexes (each reaching 13% of total isolates) (Fig 2). From the remaining 10 isolates (shown as “others”), 8 had no assigned clonal complex and 2 corresponded to the clonal complex ST-461, all of them with different sequence types.

#### Distribution of PorA subtypes

PorA sequences were obtained successfully for 182 isolates and queried for variable region classification. The nomenclature found hereafter for PorA profiles is “P1. (VR1), (VR2), (VR3)” as suggested by [31]. Despite finding a wide range of variants within each family of variable regions, we only kept the family number for analysis (Ex: P1.5–2, 2–2, 36–2 was noted as P1.5,2,36).

**Fig 3:**
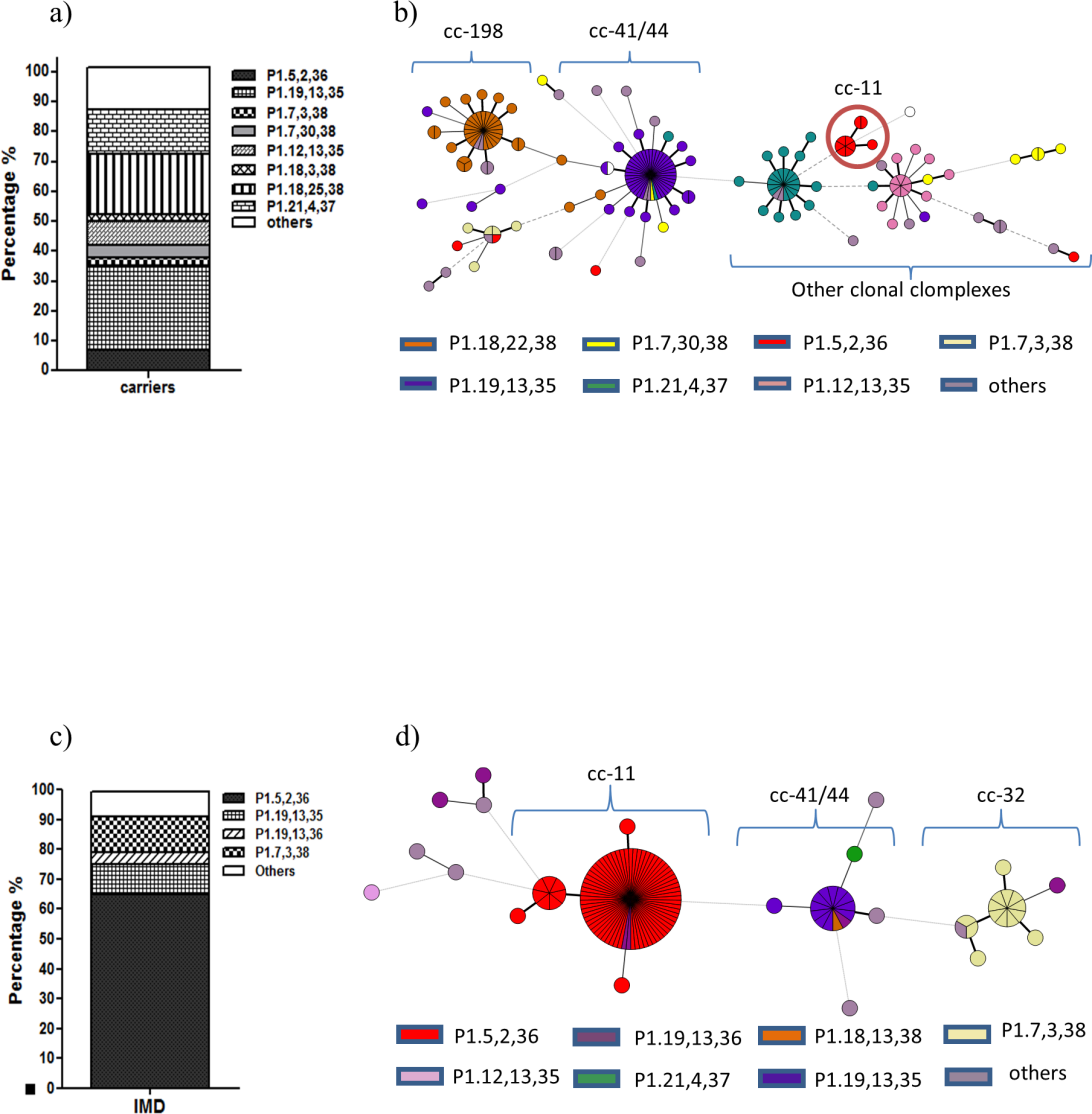
Distribution of PorA subtypes among NM isolates 2013. a) PorA profiles from carrier isolates presented as percentages b) PorA profiles from carrier isolates were differentiated by colors within clonal complexes resulting from minimum spanning tree analysis of MLST. c) PorA profiles percentages from IMD isolates. d) PorA profiles from invasive isolates indicated by colors within clonal complexes as in b). (*ND = non-determined). Compare the dominance of P1.5,2,36 subtype among invasive isolates and the diversity among carrier isolates.

As expected, a wide range of variable PorA subtypes within carriers was found (Fig 3A); whereas among invasive isolates, the subtype P1.5,2,36 (associated with strain W:cc11) was overrepresented, reaching 66% (Fig 3C). The main subtype found among carrier isolates: P1.19,13,35 (mainly associated with serogroup B) represented 28%, while the same subtype was present among 10% of invasive isolates. The PorA subtype P1.18,25,38 was found in almost 20% of total carrier isolates but was not present among invasive isolates. The subtype P1.21,4,37 reached about 15% among carrier isolates and was found only in one isolate from invasive disease during the same epidemiological year (included in Fig 3C within “others”). The subtype P1.12,13,35 reached 8% among carrier isolates while it was absent among invasive isolates. The PorA subtype P1.19,13,36 was found only among invasive isolates reaching 4%. Several other subtypes found in less than 3 isolates were grouped as “others”. It is also noteworthy, that most of PorA subtypes found within invasive isolates were also found among carrier isolates (compare Fig 3A and 3C).

When the data was arranged in a minimum spanning tree (Fig 3 panels B and D), we found a major association of PorA subtypes with each clonal complex with a few new subtypes mixed among the nodes. In particular within carrier isolates, the hypervirulent-associated subtype P1.5,2,36 (in red) was found on isolates belonging to serogroup B, Z or non-groupable that had rare or no assigned cc, in addition to the cc11 (encircled node) (Fig 3B). This observation illustrates the phylogenetic divergence of porA and the genetic transfer across different clonal complexes. Likewise, within the invasive isolates (Fig 3D), we found a predominance of the porA genotype P1.5,2,36 followed by P1.19,13,35 and P1.7,3,38. Again we found some isolates with different PorA subtypes dispersed among the nodes. For instance, the PorA subtype P1.19,13,36 (in magenta) was found among the node cc11 as well as within the nodes cc41/44, cc32 and among isolates belonging to other clonal complexes (Fig 3D).

#### Distribution of fHbp peptides

**Fig 4:**
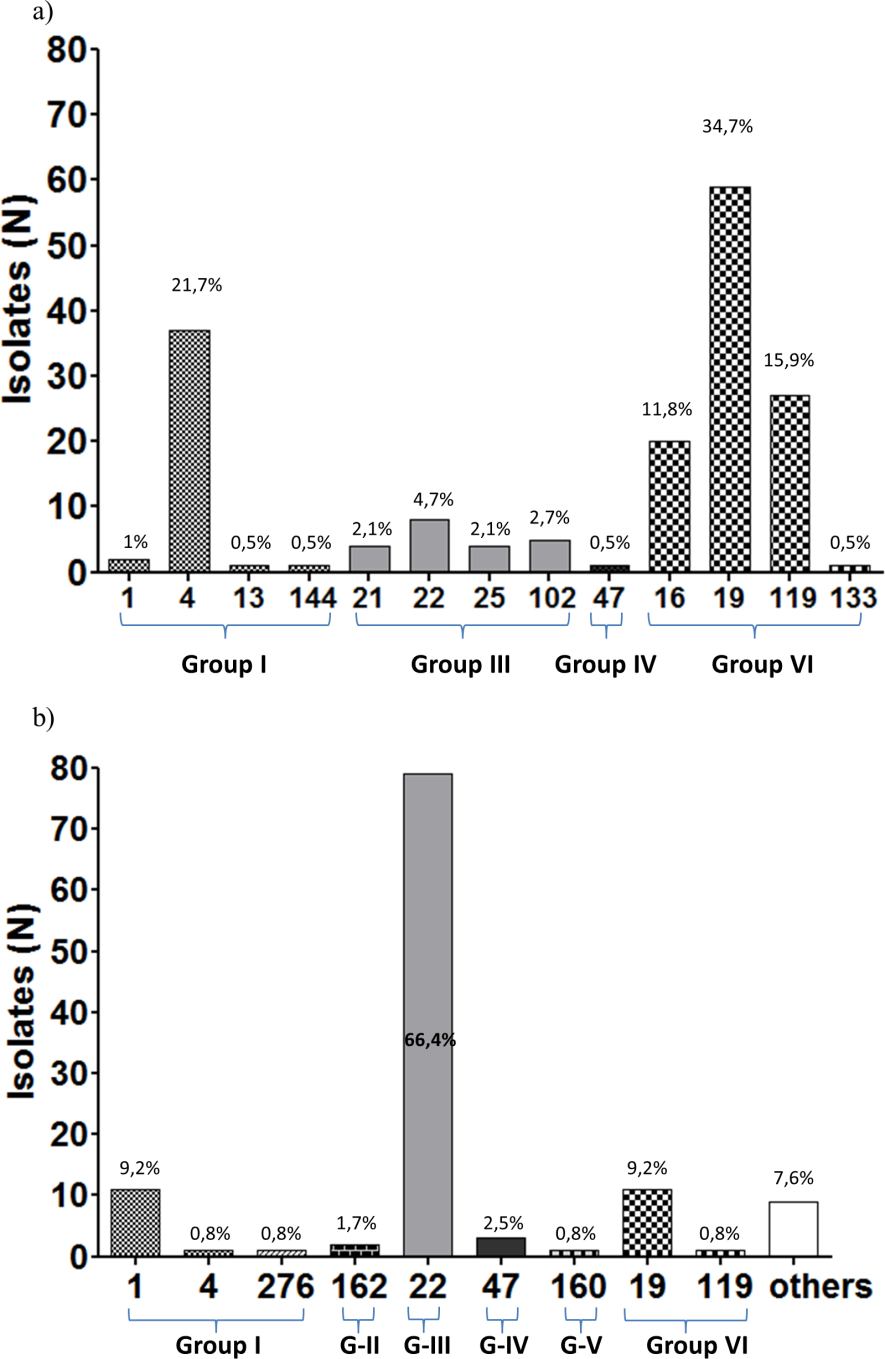
Distribution of fHbp peptides among NM isolates 2013. FHbp peptides for all isolates were arranged by modular groups as previously described [32]. Percentages from total are displayed on respective bars. a) fHbp peptides from carrier isolates (N = 184) or b) fHbp peptides from IMD isolates (N = 119). Modular group VI and I predominated among carrier isolates, whereas modular group III did among invasive isolates.

There are different nomenclatures for fHbp all based on phylogenetic analysis. Herein, we have used the typing scheme that classify sequences according to modular groups that are interchanged among variants and could share antigenic variability [32,33]. FHbp sequences were obtained successfully for 180 out of 184 carrier isolates. Within this group, we found a high diversity of peptides where the modular group VI accounted for approximately 60% of total samples; whereas the peptide 4 belonging to Modular Group I, was found in about 20% of analyzed samples (Fig 4A).

Among invasive isolates, fHbp sequences were successfully obtained for all 119 samples. Peptides were distributed along six different modular groups (Fig 4B), mainly represented by peptide 22 reaching 66.4% of total isolates and followed by peptides 1 and 19 reaching 9.2% each. Other peptides (new variants) reached 7.6%. The remaining peptides belonging to several modular groups were found in 1 to 3 isolates each.

**Fig 5:**
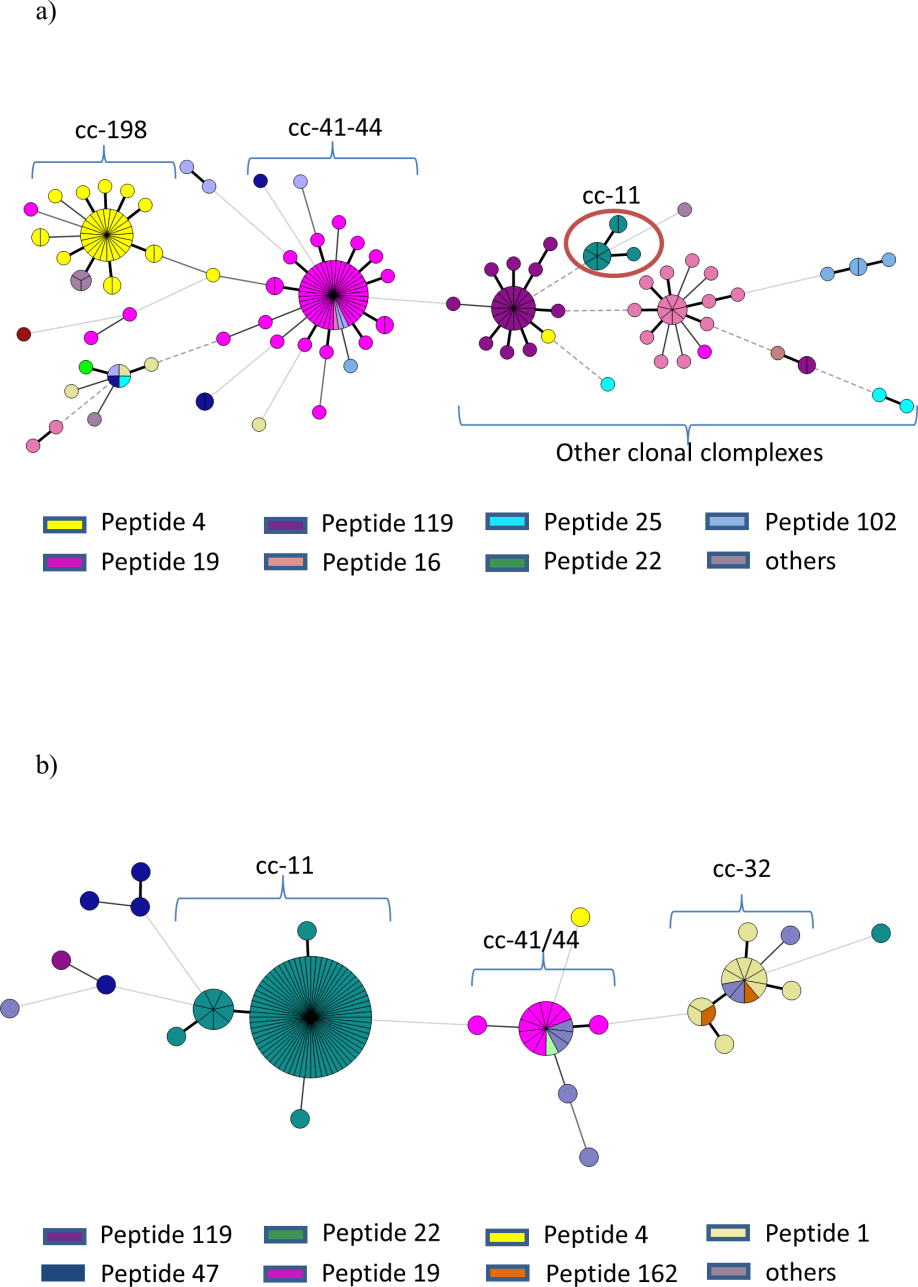
Distribution of fHbp peptides within clonal complexes of NM isolates 2013. fHbp peptides were differentiated by colors within clonal complexes arranged as minimum spanning trees. a) fHbp peptides distribution within carrier isolates (N = 184) b) fHbp peptides distribution within invasive isolates (N = 119).

In general, when arranged according to clonal complex (Fig 5), most fHbp peptides were associated with one clonal complex with less escaped variants compared to PorA (compare colors within nodes between Figs 3 and 5). In the group of invasive isolates (Fig 5B), we found an overrepresentation of the fHbp peptide 22 associated to the cc11, while the same peptide variant was found in <5% within carrier isolates equally associated to the few cc11 isolates (encircled in red).

## Discussion

Meningococcal carriage studies carried out in Chile in 2012 and 2013 have reported low rates of carriage. Data from carriers aged 18–24 years during 2012 reported 4% carriage, with only three isolates belonging to serogroup W, none of them belonging to cc11 [20]. A second study reported 6.5% carriage among adolescents aged 9–19 during 2013, with only nine isolates belonging to serogroup W (0.2% in a cohort of 4217 individuals). In this study we demonstrated that serogroup W isolates from the last carrier study belonged to the cc11. There is no proof of previous circulating hypervirulent isolates in Chile as there is in a neighboring country, Argentina, with seven isolates in 2008 [34].

The increased incidence of NM serogroup W invasive cases in Chile (66% of total IMD cases) could be a consequence of increased carriage of the same strain. This correlation has been reported previously in Burkina Faso where the predominance of W:cc11 carriage was correlated with an elevated incidence of IMD cases due to this strain during 2011 [25] and similarly among carriers from the Mecca after the W:cc11 outbreak in 2000 [35]. High carriage and disease caused by serogroup B in different countries [36], as well as increase of carriage and disease due to NM serogroup X in Burkina Faso [25,37], constitute other examples of this apparent correlation. The results shown here, however, evidence a very low carriage of W:cc11 in Chile (5% among carriers versus 66% among invasive isolates). Low carriage of this strain might be explained by an enhanced virulence to invade the host, which would allow most of colonization cases to move forward into disease. Similar low carriage rates compared to invasive cases were reported for epidemic serogroup A in Burkina Faso prior to massive vaccination [38], as well as serogroup C in the UK [39] and three other countries where serogroup C was also reported to belong to cc11, an ancestor of W:cc11 [36].

The question concerning why the W:cc11 strain described here has not triggered an epidemic as did the W:cc11 from the Hajj remains unknown. A main hypothesis is related to the genetic differences reported between these two strains [40] including fHbp (peptide 22 and 9 respectively). Another hypothesis could be related to the presence of other competing bacteria, as *N*. *lactamica*, in our populations. *N*. *lactamica*, has reduced by around 10% the carriage prevalence of *N*. *meningitidis* over at least 16 weeks by displacement and probably niche competition when inoculated [41]. Díaz et al., assessed 5% carriage of other species from the genus *Neisseria* in carrier subjects during 2013 (in addition to 6.5% carriage described for NM within the same cohort). Most of these isolates were identified as *N*. *lactamica* (105/161 cases). An additional hypothesis could be that W:cc11 in Chile might possess a reduced ability to survive outside the host, which may help to reduce the spread of disease. The last hypothesis would agree with reported survival differences among NM strains [3].

Immunization against NM has led to a reduction of IMD outbreaks worldwide [6]. Carriage reduction and herd immunity, however, are controversial, especially in countries with low incidences. Namely, in spite of low carriage, immunization against serogroups A and C in the above mentioned epidemic cases has reduced both disease and carriage [27,42,43]. However, another study has shown an increased carriage of serogroup W after MenACWY vaccination in healthy first year university students in the UK [44].

Serogroup B is the second most frequent serogroup causing disease and being carried in Chile. Vaccines against this serogroup were designed with one or several variants of bacterial OMPs. In this study we analyzed PorA and fHbp for all invasive and carrier isolates. Thus, it is partially possible to predict the potential coverage of current vaccines formulated with one or more of these OMP variants [45,46].Namely, variant 4 found within variable region 2 (VR2) of PorA, the one responsible for bactericidal antibodies in vaccines including outer membrane vesicles in their formulation [47], was not found in our invasive isolates, while it reached around 15% among PorA sequences of carrier isolates during 2013. Another vaccine, formulated with two different alleles of fHbp (peptides 45 and 55), could cover around 40% of isolates found in carriers that share fHbp characteristics and up to 18% within invasive isolates (this considering the reported cross-coverage with peptides 1 and 19). Furthermore, a recent experimental study has shown, using a functional assay, indirect protection of a serogroup B vaccine against the hypervirulent serogroup W. This protection could be mediated, by antibodies generated against NadA, NHBA or both [48]. It is not possible to discard, however, antibodies generated against other immunogen present within the OMV of this vaccine.

Furthermore, in our dataset, all W:cc11 isolates found on carriers possessed the gene encoding the peptide 22 of fHbp in addition to the PorA profile P1.5,2,36, the same as invasive isolates profile from the same year. How these carriers could resist NM invasion is unknown, but a strong immune status could be speculated as the main reason.

This study compares for the first time in Chile the NM strains found among carriers and invasive cases, during the same period. Surveillance of NM is relevant considering the emergence of new strains either by introduction from other countries or by genetic transfer among co-habiting NM species [49]. For instance, isolates from serogroup A, whose clonal complex 5 have previously caused pandemics, have not been found in Chile [50]. Similarly, concerns have been raised by the scientific community about the possible emergence of a serogroup B:cc11 strain by horizontal genetic transfer between the serogroups W and B already present in our country [51].

A limitation of this study is that carriage isolates were collected from an adolescent age group whereas IMD isolates belonged to cases of all ages. Additionally, as there is no data available, it is not possible to discard that W:cc11 strain could eventually be found in higher rates within the age groups where IMD was more elevated the same year (children within the first year of life and elderly people).

## Conclusions

The high rate of hypervirulent W:cc11 IMD cases in Chile, in a context of a general low incidence of disease, is an interesting phenomenon especially due to the very low carriage rate found for this strain during the same epidemiological year.

The observations presented in this article suggest that the strain W:cc11 found in Chile possesses an enhanced fitness to invade its host compared to other strains causing IMD in the country.

Low carriage rates have previously been reported in other countries for the strains A:cc5 and C:cc11. Nevertheless, further studies are required to understand why the strain W:cc11 described here has replaced other invasive strains in Chile without triggering an epidemic outbreak of the sort witnessed in the case of the above mentioned strains and the W:cc11 strain from the Hajj.

## Supporting information

S1 Table PCR conditions. Cycling conditions are in bold (35 cycles for PorA and 40 cycles for MLST and fHbp). All PCR amplifications were complemented with 0,1M of Betaine. We also used 0,02% v/v of DMSO for all reactions excepting fHbp. Primer concentration was 0,4 uM for fHbp and 0,8uM for all remaining alleles. *Annealing for aroE and fumC was made at 60°C (58°C for all remaining 5 MLST alleles).
(PDF)

S2 Table Serogroup distribution among carriers or IMD isolates. (PDF)

S3 Table Clonal complexes distribution among carriers or IMD isolates. (PDF)

S4 Table PorA subtypes a) Carrier isolates and b) IMD isolates.
(PDF)
