# Supplementary material for: Increase of Neisseria meningitidis W:cc11 invasive disease in Chile has no correlation with carriage in adolescents
Source: PLoS One. 2018 Mar 8;13(3):e0193572. doi: 10.1371/journal.pone.0193572 (PMC5843251; doi:10.1371/journal.pone.0193572)
Supplement: S1 Table — Cycling conditions are in bold (35 cycles for PorA and 40 cycles for MLST and fHbp). All PCR amplifications were complemented with 0,1M of Betaine. We also used 0,02% v/v of DMSO for all reactions excepting fHbp. Primer concentration was 0,4 uM for fHbp and 0,8uM for all remaining alleles. *Annealing for aroE and fumC was made at 60°C (58°C for all remaining 5 MLST alleles). (PDF) [file pone.0193572.s001.pdf]

MLST	PorA	fHbp
94°C → 5 min	94°C → 2 min	94° C → 5 min
**94°C → 1 min**	**94° C → 1 min**	**94° C → 1 min**
***58°C → 1min**	**62° C → 30sec**	**64° C → 1min**
**72°C → 1 min**	**72° C → 1 min**	**72° C → 1 min**
72°C → 5 min	72° C → 2 min	72° C → 7 min

S1 Table: PCR conditions. In bold are cycling conditions (35 cycles for PorA and 40 cycles for MLST and fHbp). All PCR amplification were complemented with 0,1M of Betaine. We also used 0,02% v/v of DMSO for all reactions excepting fHbp. Primer concentration was 0,4 uM for fHbp and 0,8uM for all remaining alleles. ***Annealing for AroE and fumC was made at 60°C (58°C for all remaining 5 MLST alleles).
