# Supplementary material for: Increase of Neisseria meningitidis W:cc11 invasive disease in Chile has no correlation with carriage in adolescents
Source: PLoS One. 2018 Mar 8;13(3):e0193572. doi: 10.1371/journal.pone.0193572 (PMC5843251; doi:10.1371/journal.pone.0193572)
Supplement: S2 Table — (PDF) [file pone.0193572.s002.pdf]

Serogroup	Carriers	IMD
B	73	38
C	33	2
W	9	79
Y	19	0
Z	2	0
*NG	48	0
**Total**	**184**	**119**

S2 Table: Serogroup distribution among carriers or IMD isolates.
