# Supplementary material for: Increase of Neisseria meningitidis W:cc11 invasive disease in Chile has no correlation with carriage in adolescents
Source: PLoS One. 2018 Mar 8;13(3):e0193572. doi: 10.1371/journal.pone.0193572 (PMC5843251; doi:10.1371/journal.pone.0193572)
Supplement: S3 Table — (PDF) [file pone.0193572.s003.pdf]

Clonal complex	Carrier (%)	IMD (%)
ST-11/ET-37	9 (5%)	78 (66%)
ST-32/ET-5	9 (5%)	16 (13%)
ST-41/44	59 (32%)	15 (13%)
ST-198	38 (20%)	0 (0)
ST-53	4 (2%)	0 (0)
ST-865	3 (2%)	0 (0)
others	58 (31%)	10 (8%)
*ND	4 (2%)	0 (0)
**Total**	**184**	**119**

S3 Table: Clonal complexes distribution among carriers or IMD isolates.
