# Supplementary material for: Increase of Neisseria meningitidis W:cc11 invasive disease in Chile has no correlation with carriage in adolescents
Source: PLoS One. 2018 Mar 8;13(3):e0193572. doi: 10.1371/journal.pone.0193572 (PMC5843251; doi:10.1371/journal.pone.0193572)
Supplement: S4 Table — PorA subtypes a) Carrier isolates and b) IMD isolates. (PDF) [file pone.0193572.s004.pdf]

a)

PorA profile	N (%)
P1.5,2,36	15 (8%)
P1.19,13,35	52 (28%)
P1.7,3,38	5 (3%)
P1.7,30,38	8 (4%)
P1.12,13,35	14 (8%)
P1.18,3,38	4 (2%)
P1.18,25,38	36 (20%)
P1.21,4,37	27 (15%)
Others	23 (12%)
	**184 (Total)**

b)

PorA profile	N (%)
P1.5,2,36	78 (66%)
P1.19,13,35	12 (10%)
P1.19,13,36	5 (4%)
P1.7,3,38	13 (11%)
others	11 (9%)
	**119 (Total)**

S4 Table: PorA subtypes. a) Carrier isolates and b) IMD isolates.
